# Availability of HIV services along the continuum of HIV testing, care and treatment in Ghana

**DOI:** 10.1186/s12913-018-3485-z

**Published:** 2018-09-26

**Authors:** Stephen Ayisi Addo, Marijanatu Abdulai, Alfred Yawson, Akosua N. Baddoo, Jinkou Zhao, Nibretie Workneh, Ivy Okae, Ekow Wiah

**Affiliations:** 1 National AIDS/STI Control Programme, Ghana Health Service, Accra, Ghana; 2 Department of Biostatistics, School of Public Health, University of Ghana, Accra, Ghana; 3 The Global Fund to fight AIDS TB and Malaria, Geneva, Switzerland

**Keywords:** Availability, HIV, Services, Continuum, Testing, Treatment, Ghana

## Abstract

**Background:**

Ghana has been providing HIV and AIDS services since the identification of the first case in 1986 and added highly active antiretroviral therapy to its comprehensive care in 2003.This study aimed at assessing availability of HIV services along the continuum of HIV care in Ghana.

**Method:**

A cross sectional study was conducted among 172 (87%) of the total 197 ART canters in Ghana. Data was collected by self-administered questionnaire and analysed using STATA version 13.

**Results:**

Of the 172 health facilities surveyed, 165 (96%) were offering HIV testing Services (HTS) during the survey period. More than 90% of the surveyed facilities reported to offer Anti-Retroviral Treatment (ART), patient counselling, TB screening and Prevention of Mother to Child Transmission (PMTCT) services. Viral load and Early Infant Diagnosis (EID) and laboratory testing services were reported at 10 (5.8%) and 23 (13.4%) respectively. HIV testing services (HTS), PMTCT, ART, patient counselling and opportunistic infections (OI) prophylaxis services were offered at all Tertiary and Regional hospitals surveyed. EID sample collection and testing services was reported at 2 out of 27 (7.4%) of the Health Centre and/or clinics in Ghana. The common adherence assessment methodology being implemented varied by facilities which included: asking patients if they took their drugs 154 (89.5%), pill counting 131 (76.2%), use of follow-up visit 79(45.9%) and use of CD4 counts, viral loads and clinical manifestation 76 (44.2%). Challenges encountered by facilities included shortage of test reagents and drugs 122 (71%), 111 (65%) respectively and patient compliance 101 (59%).

**Conclusion:**

This study showed ART services to be available in most facilities. Methods used to assess patients adherence varied across facilities. Shortage of test reagents and drugs, EID sample collection and testing were major challenges. A standardised approach to assessing patient’s adherence is recommended. Measures should be put in place to ensure availability of HIV commodities at all levels.

## Background

The National HIV/ AIDS/ STI Control Programme(NACP) was launched in Ghana in 1987 and since then there has been an increasing number of People Living with HIV (PLHIV) population as well as those put on Antiretroviral (ARVs). By the end of 2014, Ghana had an estimated PLHIV population of 226,460 of which 10.5% were projected to be children. The projected ART need was 139,157 for adults and 13,125 for children of which 73% and 63% respectively were projected to be on ART and a projected PMTCT coverage of 82% [1]. As at the end of 2014 HIV prevalence among the general population was 1.37% and 2% among ANC clients [2].

Availability of HIV-related preventive, care and treatment services is essential to reduce the burden of the disease. The continuum of HIV testing, care, and treatment covers a wide range of activities which aim at early detection of HIV cases through HIV testing services, care and support, anti-retroviral therapy (ART), adherence monitoring and follow-up, regular supply of medications, adverse event monitoring and drug resistance surveillance, and clinical, immunological and virologic monitoring [3]. However, many resource-limited countries face challenges in availing these services [4–6].

Limited or incomplete availability of services along the HIV testing- care - treatment continuum can negatively affect HIV. For example, where testing services are not available, this will undermine know your status campaign which is required to achieve the 90% of people knowing their HIV status by 2020 to achieve the end AIDS in 2030 target [3, 7]. Shortage of medications will also undermine adherence and therefore affect 90% treatment coverage required in the 90–90-90 global vision on HIV control [8].

To date, limited review has been undertaken on a range of service availability across the nation. Analyses of HIV service delivery have been fragmented with focus on only one component of the service such as ART [9], and integration of HIV and TB [10]. However, HIV service involves a continuum of integrated services and the break in this continuum can affect other components along the continuum. The concept of the continuum of care, is used to coordinate and link the health facilities, the community and other sectors under one coherent framework to ensure effective service delivery [11, 12].

Ghana operates a decentralized administrative structure with the country divided into ten regions with the Greater Accra region being the national capital of Ghana [13]. The decentralized structure follows a four-tier system; national, regional, district, and sub-district levels. Health care is also decentralized along the administrative structure with the community level serving at the first point of the primary health care system. There are also clinics and health centres at the sub-district which are often manned by nurses and headed by Physician Assistants. At the district and regional levels are hospitals which are headed by medical officers and provide the secondary level of health care. The tertiary level is provided by the teaching hospitals which are located in the Northern, Ashanti, Greater Accra, and Central regions of Ghana [14, 15]. HIV-related services are also structured along the formal health system. These facilities provide all HIV-related services and refer cases along the continuum of care based on the hierarchical structure of the health care delivery in Ghana; primary, secondary and tertiary levels [16]. Referral across the various levels is based on the competence of the service provider, availability of logistics and sometimes based on proximity of the health facility as prescribed in Ghana ART guidelines. [17] With respect to client load per facility, the tertiary and regional hospitals have most clients as compared to the other facility types.

Ghana has been providing HIV and AIDS services since the identification of the first case in 1986, adding and expanding gradually services along the continuum over the past decades. The provision of the highly active antiretroviral therapy (HAART) was added to its comprehensive care in 2003. Ghana’s Prevention of Mother to Child Transmission (PMTCT) program started effectively in 2002 with two facilities offering PMTCT services in the Manya Krobo District in the Eastern Region. Since then, the country has expanded PMTCT facilities to 2748 of over 3765 health facilities providing Antenatal services. Syphilis testing is also offered to all the pregnant women visiting the PMTCT centers. This study was to cross-sectionally assess availability of HIV services along the continuum of HIV care in Ghana as a step towards improving the quality of HIV and AIDS service delivery in the country.

## Methods

### Study design

This was a cross-sectional survey which employed the administration of questionnaires to frontline service providers with at least 1 year experience in the area of HIV service delivery.

### Study participants

The respondents for each facility were health care providers directly involved in the provision HIV related services in their respective facilities for at least 1 year. They were made up of clinician’s nurses, midwives, data managers, pharmacist and laboratory personnel who formed the core ART team at their various facilities.

### Study area

We conducted our study in Ghana which is located in the West African sub-region. The country is bordered to the south of Gulf of Guinea and the Atlantic Ocean and is bordered to the west by Cote d’Ivoire, east by Togo and Burkina Faso to the north. The population of the country is estimated to be 27,000, 000 based on the 2010 Population and Housing Census Report [18].

### Data collection

A health facility assessment one pager questionnaire was administered to all 197 ART sites in Ghana (Total audit). Out of which one hundred and seventy-two sites (87%) responded to the questionnaire with the remaining sites opting out. The indicator selection for the questionnaire was guided by WHO’s guide to monitoring and evaluation of HIV/AIDS care and support [19]. This survey was conducted to document the availability and quality of ART services from service providers’ perspective. Data gathered included information on the services that they offer at that particular facility, flow of the services at ART initiation and during the follow up visits, ART adherence assessment methodologies, management of ART patients in and out transfers, management of lost to follow up and use of the clinic routinely collected data. Also the questionnaire gathered information on challenges faced by both service providers and ART clients. These interviews took place at the facilities between 2015 and 2016. Data collectors were the National AIDS/STI Control Programme (NACP) data officers. They were trained on the contents of the survey tools and survey procedures. Only one questionnaire was completed per facility.

### Ethical issues and confidentiality

The study was approved by the Ethics Review Committee (ERC) of the Ghana Health Service. Permission was sought from Regional and District Directors of Health services as well as facility heads involved in the study. Informed consent was also sought and obtained from all participants. They were assured of their privacy and confidentiality. No personal identifiers were captured during data collection. The respondents were informed that they could opt-out of the study at any point and were also not obliged to respond to all the questions. Data collected was kept in a locked shelve at the secured NACP records room.

### Data analysis

The questionnaire was coded, double entered into Microsoft (MS) access by two independent data entry clerks and later transferred to Stata data analysis software for data management and analysis. Descriptive statistical method was used to present proportions of variables of interest.

## Results

### Composition of facilities assessed

**Fig. 1:**
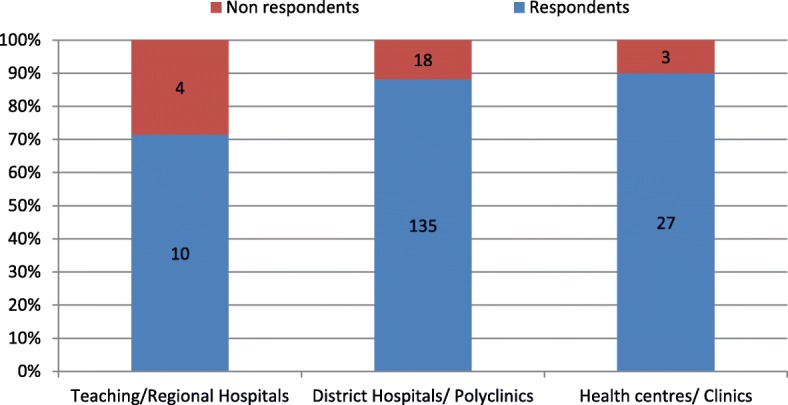
Respondent and Non- respondents by Type of Health facilities

In total, one hundred and seventy-two health facilities responded to the questionnaire (87%). The type of facilities that responded included teaching hospitals (*n* = 2); Regional hospitals (*n* = 8); District hospitals (*n* = 131); Polyclinic (*n* = 4); Health centres (*n* = 19) and clinics (*n* = 8). However, these were later during the analysis re-categorised into three groups; teaching and regional hospitals in one group, district hospitals and polyclinics in a second group and health centres and clinics in the third group. The facilities that did not respond to the questionnaire were teaching hospitals (*n* = 2); Regional hospitals (*n* = 2); District Hospitals (*n* = 17); Polyclinic (*n* = 1); Health centres (*n* = 2) and clinics (*n* = 1) Fig. 1 provides proportions of both respondents and non-respondents to the survey.

### Routine HIV related service offered at different ART facilities

Service providers were asked about HIV related services that were offered at their facilities. Services that were offered at different facilities ranged from HIV testing and counselling to viral load specimen collection and laboratory testing. About 95.9% of the surveyed facilities were offering HIV Testing and Counselling (T&C) services. More than 90% of the surveyed facilities reported to offer ART patient counselling, TB screening and PMTCT services On the other hand, viral load and EID specimen collection and laboratory testing was done at only 10/172 (5.8%) and 23/172(13.4%) of all survey facilities respectively because there are only nine viral load machines located in regional/teaching hospitals. All facilities in a region share the viral load machine. Table 1 shows the services provided by the health facilities.

**Table 1:** Distribution of services provided across various health facilities

Type of Health facility	Tertiary Regional Hospitals (*N* = 10)		District Hospitals and Polyclinics (*N* = 135)		Health Centre Clinics (*N* = 27)		Total	
Services offered	n	%	N	%	N	%	N	%
ANC	9	90.0	117	86.7	20	74.1	146	84.9
PMTCT	10	100.0	122	90.4	23	85.2	155	90.1
HIV TC	10	100.0	129	95.6	26	96.3	165	95.9
ART Patient Counselling	10	100.0	129	95.6	23	85.2	162	94.2
ART Patient Peer Support	9	90.0	81	60.0	8	29.6	98	57.0
CD4 Specimen	3	30.0	32	23.7	10	37.0	45	26.2
CD4 Specimen + laboratory	8	80.0	83	61.5	9	33.3	100	58.1
TB Screening	9	90.0	124	91.9	24	88.9	157	91.3
Viral Load Specimen	4	40.0	41	30.4	6	22.2	51	29.7
Viral Load Specimen + lab	5	50.0	5	3.7	0	0.0	10	5.8
EID Specimen	4	40.0	96	71.1	10	37.0	110	64.0
EID Specimen + Lab	6	60.0	15	11.1	2	7.4	23	13.4
STI Diagnosis + Treat	9	90.0	109	80.7	17	63.0	135	78.5
OI Prophylaxis	10	100.0	120	88.9	21	77.8	151	87.8

### HIV related service by type of facility

The study found that PMTCT, HIV Testing & Counselling, ART patient counselling and Opportunistic Infections(OI) prophylaxis services were offered at all Tertiary and Regional hospitals that were surveyed. Furthermore, TB screening was reported to be offered at majority of the different type of health facilities that were surveyed. More interestingly we found that Early Infant Diagnosis (EID) sample collection and testing was done at 2 out of 27 (7.4%) of the Health Centre and/or clinics in Ghana.

### Methods used to assess adherence to HIV treatment by health facilities

**Fig. 2:**
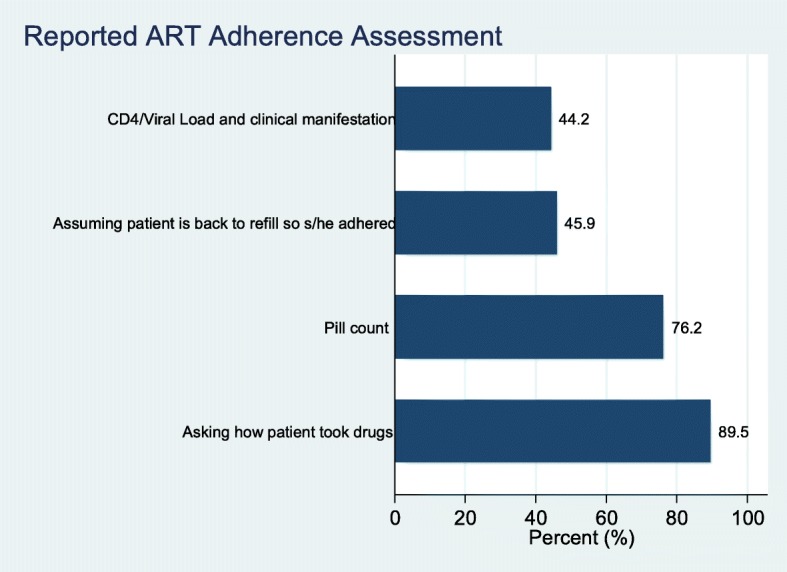
Reported methodology for assessment of ART adherence

Various ways of assessment of adherence to HIV treatment were reported at different facilities. The commonly reported methodology of assessing adherence was by asking the patient to explain how s/he took the medication and this was reported at 89.5% of all the facilities that participated in this survey and pill counting methodology was reported at 76.2% of the facilities that were visited. In addition, about 45.9% of the respondents revealed that adherence was assessed on assumption that since the patient came back for refill that implied that s/he adhered. Furthermore, staff at 44.2% of the surveyed facilities reported that they assess adherence to HIV treatment based on patient’s CD4 count, viral load or clinical manifestation (Fig. 2).

The study further disaggregated data to identify the methods used to assess adherence by type of health facility. The results showed that the most common method that is employed across all facilities was asking the patient if they had taken their medication. Pill count was the second common method used by various health facilities. The methodologies for assessing patient were slightly different when comparing different type of facility and the difference was statistically significant.

### Management of ART patients in and out transfers

**Fig. 3:**
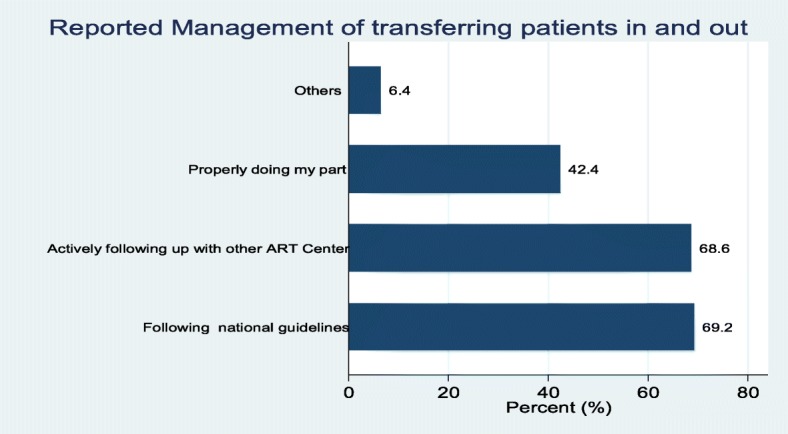
Management of ART patients in and out transfers

Transferring patients in and out of ART clinics is a common practice in ART clinics in Ghana. During this survey, health care providers were asked to describe how they manage transfers in and out for ART patients. Respondents at about 69% of the surveyed facilities reported that this was done by following the national guidelines and the same proportion reported that this was done by actively following up and tracing the patient at the other ART centres. Respondents at about 42% of the facilities indicated that they manage the transfers of patients by properly doing their part (What they think is right) (Fig. 3).

Furthermore, the findings were stratified by the type of health facility and we found that the proportion of the facilities reporting to manage the transfers by actively following up with other ART clinics and by following the national guideline was slightly different when compared by type of facility, however these differences were not statistically significant (*p* = 0.784 and 0.667 respectively).

### Management of ART patient’s lost to follow up

**Fig. 4:**
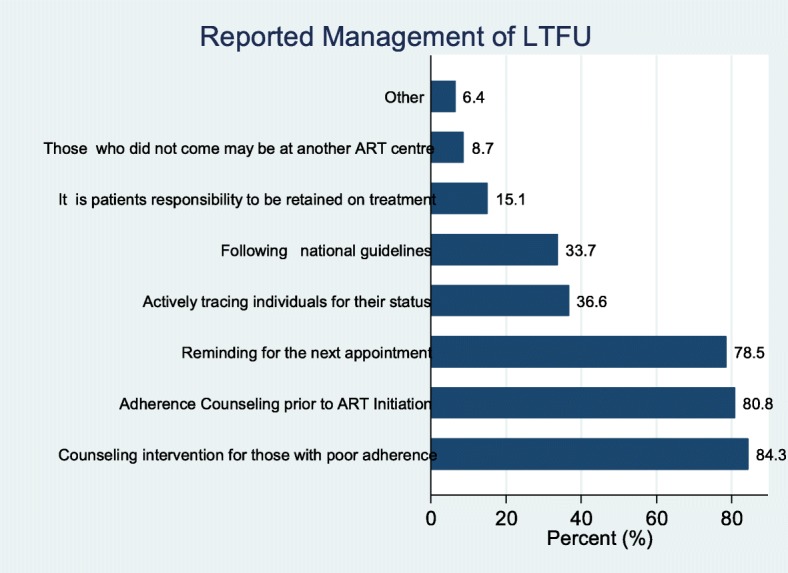
Management of ART patients LTFU

Patient Loss to follow up (LTFU) poses challenges to the successful implementation of ART programs. During the health facility survey, service providers were required to describe how they prevent or manage LTFU at the facility level. About 84% of all facilities that were surveyed, care providers indicated that patient LTFU was minimised by identifying patients with poor adherence to ART and providing special counselling interventions. Service providers at 81% of the survey facilities reported that LTFU is managed or prevented through proper counselling of HIV patients on adherence prior to initiation on treatment. Furthermore, service providers at about 78% of surveyed facilities indicated that LTFU is prevented by actively reminding the patients of their next appointment date through phone calls. On the other hand, service providers at about 15% of the surveyed facilities were of the view that retention on treatment is the patients’ responsibility and thus were a personal decision while about 9% of the service providers interviewed reported that sometimes those who do not attend to their appointment dates might have gone to another ART clinic (Fig. 4).

These findings were further stratified according to the facility type/level and we found that the reported methodologies used to prevent LTFU in ART clinics were similar across different facility level.

### Service providers’ reported daily challenges

**Fig. 5:**
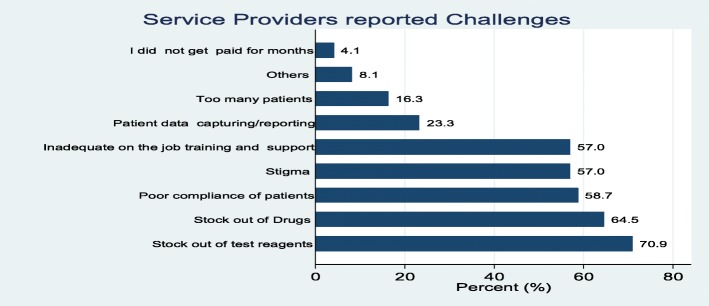
Service providers’ challenges

Service providers were asked of the challenges they encountered while working at ART clinics. The challenges mentioned in the various facilities included shortage of test reagents at about (71%), shortage of drugs (65%) and patient compliance (59%) of the facilities. Others included inadequate on the job training and support (57%), stigma (57%), patient data capturing and reporting (23%) and high number of patients (16%). Figure 5 provides a summary of the challenges identified in the study.

**Fig. 6:**
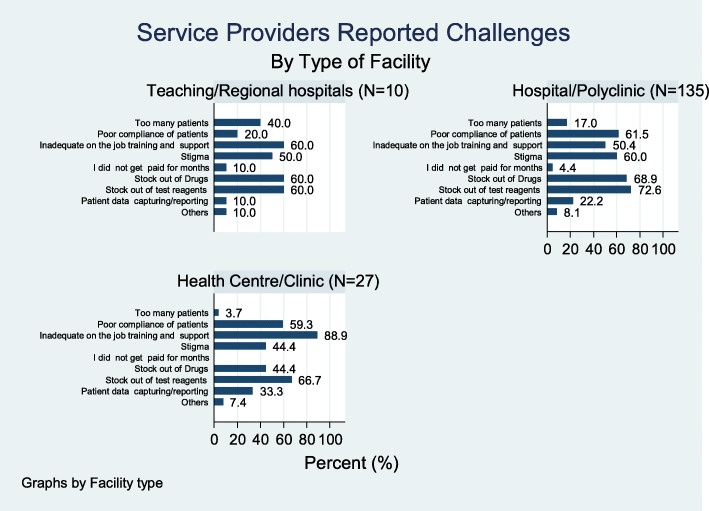
Service providers challenge by type of facility

Service providers’ reported challenges were further stratified by the level of the facility and we found that services providers at the Teaching and Regional hospitals were more likely to report large number of patients to be a challenge compared to other facilities though the difference was not statistically significant (40% vs.14%; *p* = 0.113). A high proportion of respondents at the health centres and clinics reported on-the-job training and support to be a challenge when compared to respondents at teaching, regional and other hospitals (89% vs.51%; *p* = 0.05). Shortage of test reagents and stock out of drugs was reported similarly across different types of health facilities (Fig. 6).

### Use of routine clinic data

**Fig. 7:**
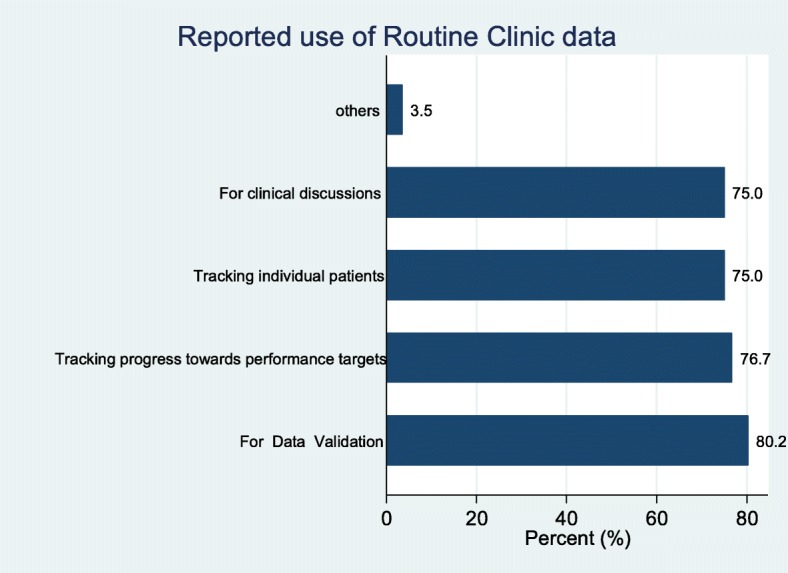
Use of routine clinic data at facility level

A broad range of data is collected routinely at the ART clinics in Ghana. During the facility survey, service providers were asked to describe how they use the routinely collected ART clinic data. At 80.2% of the surveyed facilities, respondents reported that routine data was used for data verification (validation). Other reported uses of the clinic routine data at facility level included tracking progress towards facility performance targets (76.7%), for tracking patients (75.0%) and for clinical discussion (75.0%) (Fig. 7). Reported use of data was similar at the different level of health facility.

### Challenges commonly reported by ART clients

**Fig. 8:**
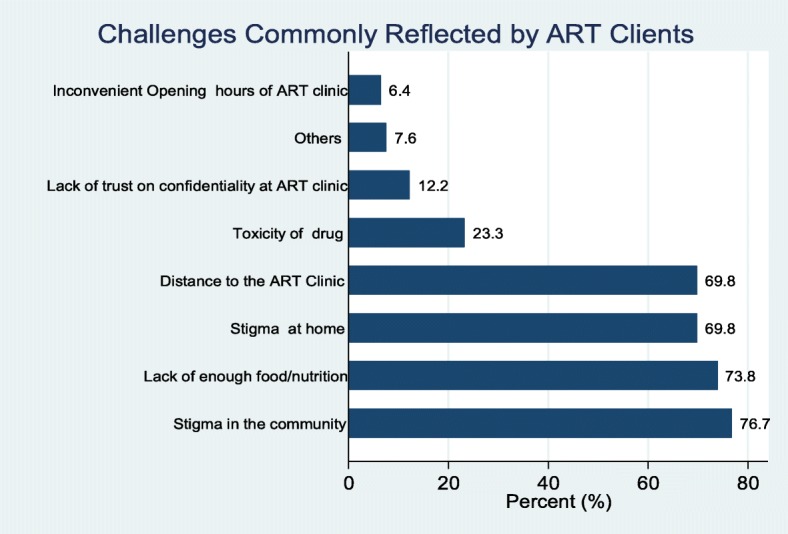
Challenges commonly reported by ART clients

When service providers were asked about the challenges commonly reported by HIV patients who are initiated on ART and attending at the survey facilities, 76.7% reported stigma in the community and 69.8% reported stigma at home to be common challenges they encounter. Lack of food and/or nutrition was mentioned at 73.8% of the surveyed facilities and distance to the ART clinics was mentioned at 69.8% of the surveyed facilities (Fig. 8).

## Discussion

This cross-sectional survey, which had respondents from a representative number of ART centres in Ghana, sought to assess the availability of HIV services across the continuum of care in the country. Findings from this survey can be generalised to all ART sites in Ghana during the study period. The findings, as above, showed availability of services for HIV testing, treatment and retention in care across various levels of care in Ghana. The gaps identified and their implication on attainment of the 90–90-90 targets for the country is discussed, and suggestions for improvement proposed. Also discussed are the challenges faced by the service providers and their ART clients, as well as the use of their routinely collected data.

Apart from EID that had low coverage in all the surveyed facilities and Viral load testing that was low due to the regional distribution of logistics, the coverage for the other testing services(HTC, PMTCT) was generally high(> 90%) among the responding facilities during the study period. The high coverage of HTC and PMTCT is a positive indicator towards the attainment of the first 90. Other approaches such as the adoption of Provider initiated testing and counselling proposed by WHO in 2007 will need to be employed to help increase the yield of testing in facilities. Despite the small number of viral load testing sites, which is similar in other resourced limited settings [20] the low proportion of facilities that take samples for EID, CD4 and viral load testing is a major barrier to reduction in mother to child transmission, and an objective assessment of patient adherence and clinical improvement on ARVs. This will adversely affect the timely enrolment of infected exposed children into care as evidenced in Ghana’s 30% EID coverage in 2015 and therefore hamper the attainment of the first and second 90s. It will also affect the attainment of the 3rd 90 because those on treatment who are virally suppressed can’t be objectively documented.

Again, ART patient counselling, ART patient peer support, TB Screening and OI prophylaxis were the support services assessed by this survey. Apart from a little over half of the facilities (57%) providing peer support, the other services were largely available in the surveyed facilities. This is good for the attainment of the 3rd 90 because these services have been shown to improve patient retention on treatment, and ultimately viral suppression [21]. The challenges making it difficult to get these services in all facilities needs to be assessed and curbed, in order to improve the quality of life and eventually reduce the epidemic through viral suppression.

The survey looked at assessment of adherence, management of patient transfers and management of client loss to follow up. From the findings it could be seen that subjective methods (Patient self-report and pill count) were largely used to assess adherence instead of objectively using viral load, CD4 counts and clinical manifestation as recommended by WHO. Although, the study found that majority (89.5%) of health facilities rely on patients self-report to determine adherence to treatment. it is important to note that self-report may not always be accurate especially in a situation where a patient thinks he/she may be scolded if they indicate that they had defaulted taking medication. It would therefore be important to complement this method with pill count and CD4 count which may be more accurate. Good health status measured by blood chemistry has also been found to correlate with adherence [22]. Combining patients recall, pill count and patients’ blood chemistry has been found to be a more accurate way of determining adherence than a single method [23]. In this study 76.2% of facilities rely on pill count only while 44.2% used CD4 count. An earlier study also found that unannounced pill count and 3-days visual analogue scale were good measure for determining adherence as they have positive reflection on CD4 [24]. Hence where pill count is to be used it should be unannounced and this is more appropriate during home visit than when the patients visit the health facility. Ghana could also explore the possibility of adopting the visual analogue scale. As similar scale was also found to be effective in a study conducted in China [25].

The study findings also showed that 69% of the facilities that participated in this study handle transfer in and out according to national guidelines. This is quite worrying as it will negatively affect the 2nd and eventually the 3rd 90 in the 90/90/90 global targets because adherence to treatment can’t be assured if the movement of clients is not organised.

This therefore underscores the need for training of service providers in this direction. Adherence to national guidelines is essential to track progress of patients as well provide good data for decision-making. In the absence of uniformity on how patients transfer in and out is managed, it would be difficult to compare data across facilities. Uniformity in data is basis for global comparison of performance of indicators [26, 27].

Furthermore, the study found that counselling was the main strategy used to ensure adherence by patients as this was reported by 84% of the facilities that participated in the study. About 78% of the facilities also remind patients of their next appointment day. However, about 15% of the surveyed facilities felt that it was the responsibility of the patient to adhere and hence nothing was done to ensure they adhere to treatment. This is disturbing as it might hinder the attainment of the 2nd and 3rd 90 in the 90–90-90 global agenda. It is therefore important that NACP puts in place measures to ensure all facilities stick to the national guidelines for tracking patients. Although counselling on the need to adhere to treatment may be useful especially when it is individualized [28], in some settings counselling alone has been found to be less effective [29]. To that in recent times, countries have been exploring a better way to improve adherence to ART. Text messages and web-based application to reminding patients [30, 31] and use of visualized mobile application to provide information to clients on their progress have been found to be effective in recent time [32]. Ghana could also explore how to adopt this strategy since mobile penetration is high [13].

The study also highlighted major challenges in HIV-related service delivery. These include logistical challenges such as shortage of test reagents (71%), shortage of drugs (65%), patient adherence (59%), lack of training and support (57%), stigma (57%), patient data capturing and reporting (23%) and high number of patients (16%). With stock out of drugs and reagents occurring in majority of the survey facilities, attainment of all the UNAIDS 90/90/90 will be significantly affected.

These Shortages can be minimized through good logistical management system. Hence there will be the need to train health facilities on how to keep stock and stock management as this has been found as a forum to improve stock keeping [33]. If this is not done, it will adversely affect control of HIV. This is because if patient report to health facilities to be tested for HIV and there are no test kit, then the health system will miss opportunities to screen people. Shortage of medication will also negatively affect adherence and it associate effects such as drug resistance, risk of transmission to people and HIV-related morbidity and mortalities [34–36]. Distance to the facility, stigma at home, lack of enough food/ nutrition and stigma in the community were the major challenges reported by clients in at least 69% of the surveyed facilities. These are likely to affect adherence and compliance to ART and thus the second 90, and eventually the third 90 when such non-compliant patients are not virally suppressed.

Of all the uses that the facilities put their data to, none of the survey facilities stated the use of the data to monitor and support supplies chain management. This might be responsible for the stock out of essential commodities.

## Conclusion

This study showed ART services to be available in most facilities. Methods used to assess patients adherence varied across the health facilities. Shortage of test reagents and drugs, EID sample collection and testing came out as major challenges. With these identified challenges confronting Ghana as a resource limited country compared to other well-resourced countries, Ghana will need to put in-place concerted efforts to be able to achieve the UNAIDS 90/90/90 of ending AIDS by 2030. Measures need to be put in place to ensure availability of HIV commodities at all levels. A standardised approach to assessing patient’s adherence to treatment is recommended in all health facilities. Again the country needs to come out with a plan to scale up viral load and EID sample collection and testing at most facilities in the country.

### Study limitation

The study did not collect data on stock out related to all HIV commodities. How long these stock outs normally last and how sites manage in these situations. A qualitative research may be required in future to help identify factors that may be responsible for the shortages.

## Abbreviations

ART: Anti-retroviral treatment
EID: Early Infant Diagnosis
LTFU: Loss to follow up
NACP: National AIDS Control Programme
OI: Opportunistic Infections
PMTCT: Prevention of Mother to Child Transmission
